# Automatic Saliva Ejector

**Published:** 1874-02

**Authors:** 


### AUTOMATIC SALIVA EJECTOR.

This instrument, the principle of which has been long known, and used for various other purposes, has recently been arranged and applied to the purpose indicated by the caption of this notice, by Dr. J. E. Fisk, of Salem, Mass.

We have had it in use for about a month, and it accom plishes the work intended as perfectly as could be desired. The application of the instrument is without any inconvenience whatever; is not in the way of any operation upon the teeth; and in any case, it matters not how copious the flow may be, it removes all the saliva as fast as it accumulates. It affords very great relief to to both operator and patient in all protracted operations where the usual excess of saliva is a great nuisance. This with the rubber dam gives complete and comfortable control of any case, so far as saliva is concerned. When its value is known, it will necessarily supersede the entire list of saliva pumps. Running water is requisite for its use, but a small amount is sufficient, and almost anywhere hydraulic arrangements can be improvised quite sufficient for the purpose. This is an appliance so valuable that no dentist can afford to be without. It is not expensive, the price is $15.00. The immunity which it gives from annoyance from excessive flow of saliva will in a few cases far more than compensate for its cost. If any one does not believe this statement, we shall insist upon his trying the instrument.

